# Clozapine-resistant patients: new treatment lines in schizophrenia

**DOI:** 10.1192/j.eurpsy.2026.10528

**Published:** 2026-07-31

**Authors:** J. Gimillo Bonaque, C. D. Mayoral, M. S. Molina

**Affiliations:** Psychiatry, Hospital Univeristario Príncipe de Asturias, Madrid, Spain

## Abstract

**Introduction:**

Treatment resistant schizophrenia (TRS), the lack of response to at least two antipsychotics administered at adequate dose and duration, epitomizes in psychiatry one of the most difficult-to-treat pathologies, epidemiologically relevant (affecting one-third of schizophrenia patients) and with severe consequences for the patients in terms of overall functioning. After 50 years, only one drug is approved for TRS: clozapine. Furthermore, a few patients do not respond even to clozapine and are indicated as clozapine-resistant patients.

**Objectives:**

Review the different new treatments of clozapine-resistant patients, **seeking to clarify who also offer more quality of life** with **few adverse effects**.

**Methods:**

In this review and expert opinion, we have critically appraised the current literature, discussing the role of old and new agents in treating resistant schizophrenia, analysing articles published in the last few years.

**Results:**

The search for therapy against TRS, beyond clozapine or in addition to clozapine, has emerged over time, capturing mainly three types of strategies: 1. Add-on of a second-generation antipsychotic (i.e. amisulpride); 2. Add-on of a second antipsychotic with significantly different receptor profile compared to the older ones (e.g. aripiprazole and cariprazine); 3. Novel strategies beyond dopamine D2/D3 receptor occupancy (e.g. xanomeline + trospium, TAAR1-agonists, sodium benzoate, and D-amino acids).

**Conclusions:**

More high-quality clinical trials applying the current operationalized criteria for TRS and clozapine-resistance are required to evaluate the efficacy of alternative and add-on treatments. There seems, now there are more strategies of intervention in that type of patients in comparation with the last ten years.

**Disclosure of Interest:**

None Declared

